# THE LTSS STATE SCORECARD AND THE ROLE OF STATE POLICY

**DOI:** 10.1093/geroni/igad104.0591

**Published:** 2023-12-21

**Authors:** Hemi Tewarson

**Affiliations:** National Academy for State Health Policy, Washington, District of Columbia, United States

## Abstract

States play a crucial role in long-term services and supports. State agencies and legislatures have the power to make LTSS and family caregiver supports more (or less) accessible, affordable, and high-quality for the residents they serve. In addition, much of the policymaking during and after the peak of the COVID-19 pandemic took place at the state level. State agencies had the authority to grant providers, consumers, and family caregivers new flexibilities and options as it relates to LTSS, and states have and continue to decide where much of the federal COVID-19 funds (e.g., American Rescue Plan HCBS funding) will ultimately go. This portion of the symposium will present an overview of where states are today with LTSS and how the Scorecard can help both identify and drive system change.

